# Roux-en-Y gastric bypass in rat reduces mu-opioid receptor levels in brain regions associated with stress and energy regulation

**DOI:** 10.1371/journal.pone.0218680

**Published:** 2019-06-20

**Authors:** Matthew McGregor, John Hamilton, Andras Hajnal, Panayotis K. Thanos

**Affiliations:** 1 Behavioral Neuropharmacology and Neuroimaging Laboratory on Addictions, Clinical Research Institute on Addictions, Department of Pharmacology and Toxicology, Jacobs School of Medicine and Biosciences, State University of New York at Buffalo, Buffalo, NY, United States of America; 2 Department of Psychology, State University of New York at Buffalo, Buffalo, NY, United States of America; 3 Department of Neural and Behavioral Sciences, The Pennsylvania State University College of Medicine, Hershey, PA, United States of America; Western University of Health Sciences, UNITED STATES

## Abstract

Roux-en-Y gastric bypass surgery (RYGB) is the most common and effective weight loss procedure for severe obesity. However, a significant increase in addictive behaviors and new-onset substance use disorder (SUD) are sometimes observed post-surgery. The endogenous opioid system is known to play a major role in motivated behavior and reward, as well as the abuse of substances, including alcohol, tobacco, opioids and highly palatable foods. Here, we examined the effects of RYGB on mu-opioid receptor levels in the brain. Male Sprague-Dawley rats were assigned to one of four groups: standard diet with sham surgery (control), *ad libitum* high-energy high-fat (HF) diet with sham surgery, calorie restricted HF diet with sham surgery (Sham-FR), or HF diet with RYGB surgery. Control and HF groups were fed their respective diets for 8 weeks, with surgery performed on the eighth week. After 9 weeks on their respective diets post-surgery, animals were sacrificed for mu-opioid receptor autoradiography using the [^3^H] [D-Ala2,N-Me-Phe4-Gly5-ol]- enkephalin (DAMGO) ligand. Rats with RYGB showed reduced DAMGO binding in the central amygdala compared to sham-operated HF diet controls, and in the hypothalamus compared to high-fat fed Sham-FR. Diet alone did not change [^3^H] DAMGO binding in any region. These findings show that RYGB surgery, independent of diet or caloric restriction, decreases mu opioid signaling in specific regions important for stress and energy regulation. Thus, RYGB surgery may lead to greater stress sensitivity via downregulated mu opioid signaling in the central amygdala, which may contribute to the observed increased risk in some subjects for addictive behavior.

## Introduction

For the past 30 years, Roux-en-Y gastric bypass surgery (RYGB) has remained one of the most common and the most effective surgical procedure in use for treating obesity and its comorbidities [1, 2]. By decreasing the size of the stomach and bypassing parts of the proximal small intestine, the surgery decreases the amount of food a patient can consume at any one time, restricting meal-size and discouraging binge eating [3]. However, some patients who have undergone RYGB surgery also see a significant increase in addictive behaviors [4], including gambling [5] and new-onset substance use disorder (SUD), such as misuse of alcohol [6–8] cigarettes [9], and opioids [10, 11]. Increased alcohol consumption [12–15] and morphine self-administration [16] have also been demonstrated preclinically in rats that have undergone RYGB surgery suggesting that biological mechanisms play a major role. However, the actual mechanisms underlying the emergence in SUD following the surgery remain unknown.

The mu opioid receptor (MOR) is associated with reward and addictive behaviors. Cocaine dependence is positively correlated with MOR expression in the cingulate, frontal and temporal cortex, caudate, and thalamic areas of the reward circuit [17], and alcohol dependence is positively correlated with MOR expression in the ventral striatum and nucleus accumbens [18]. The effects of opioid use on MOR expression is inconclusive, with some studies showing downregulation and others upregulation in these reward areas [19]. Moreover, the MOR is present in nearly all brain areas associated with feeding, including those involved in both the energy regulation and reward aspects of food consumption [20]. Long-term exposure to a palatable diet has been shown to induce changes in MORs in the mesolimbic dopamine circuit [21]. MOR mRNA has been shown to decrease in the ventral tegmental area and nucleus accumbens in high-fat diet fed mice compared to standard (low fat) diet controls [22, 23]. When a high-energy, high-fat/high sugar diet is restricted to match caloric intake of rats eating standard chow, there is no difference in MOR mRNA levels in the nucleus accumbens, suggesting increased calorie intake or obesity is necessary to induce changes in MOR availability, not the macronutrient composition of the diet [24].

Injection of MOR agonists increases the intake of palatable foods regardless of caloric value, indicating that they are important to the rewarding aspects of consumption [20]. The MOR agonist D-Ala2,N,Me-Phe4, Gly-ol5-enkephalin (DAMGO) has been implicated in the increased preference for a high-fat diet [25]. This effect is especially apparent in the nucleus accumbens, where stimulation with DAMGO triggers an increase in food intake even when sated [26]. Injection of DAMGO also increases motivated behavior for a sweet food reward, inducing increased lever pressing for sugar cubes in rats [27]. Additionally, increased DAMGO binding in several brain regions is associated with consumption of a highly palatable diet, both with and without corresponding weight gain [28]. RYGB has been shown to reduce preferences for highly palatable sugars and fat in rats, an effect that had been initially attributed to altered reward functions [29, 30], while more recent works in rats and human debate that notion [31, 32]. Moreover, RYGB surgery in HF diet-fed rats has been demonstrated to reduce brain MOR availability in the hypothalamus, amygdala, and striatum as measured by [^11^C]carfentanil PET imaging, and reduce MOR protein expression in the striatum and prefrontal cortex, compared to those receiving sham surgery [33] These changes in opioid signaling were accompanied by decreased HF diet intake and preference in the RYGB group.

Recently, our laboratories reported that changes in the dopamine system induced by a high-fat diet are reversed by RYGB [34]. Dopamine receptor binding decreased in the striatum and nucleus accumbens of rats fed a high-fat diet, while RYGB rats showed no difference in binding compared to standard diet controls, indicating that RYGB reverses the dopamine system changes induced by the high-fat diet. In the present study, we seek to examine the mu opioid receptor levels throughout the brain following RYGB surgery, and whether these changes are the result of caloric restriction or an effect of the surgery itself. We expect that a high-fat diet will increase mu opioid receptor expression in regions of the reward circuit, and that RYGB might similarly reverse this effect.

## Methods

### Animals

Four-week old male Sprague‐Dawley rats (Charles River, Wilmington, MA) were used in this study, and were placed on either a high-fat (HF) diet (60% kcal from fat; D12492, Research Diets, Inc., New Jersey) (*n* = 28) or normal diet (“ND”, “Control”) diet (13.5% kcal from fat; Purina Mills, Missouri) (*n* = 9), which was maintained for the duration of the study. Animals were individually housed in a humidity-controlled vivarium on a 12-hour light-dark cycle (lights on at 0700h), and had *ad libitum* access to water and their respective diets. After 8 weeks on the designated diet (12 weeks of age), the HF diet‐induced obese rats received either RYGB (*n* = 9), or sham surgeries (*n* = 19), consistent with previous studies [14]. The surgical techniques, pre- and postoperative care were carried out as described elsewhere [35]. Of the sham‐operated rats, 10 received a HF diet and water *ad‐libitum* (“Sham High-Fat”, “Sham-HF”) while 9 received *ad libitum* water but were pair‐fed the previous daily average of HF diet consumed by the RYGB group (“Sham High-Fat FR”, “Sham-FR”). The control group (n = 9) did not receive surgery, and maintained a normal diet. At 21 weeks of age all rats were deeply anesthetized using 3% isoflurane and sacrificed via decapitation. Brains were then harvested and flash frozen in 2-methylbutane and stored at -80°C. Body weights were monitored throughout the experiment, and food intake was not recorded regularly as weight outcomes were the primary objective of the study. All experiments were conducted in conformity with the National Academy of Sciences Guide for Care and Use of Laboratory Animals, and approved by the Pennsylvania State University College of Medicine and University at Buffalo Institutional Animal Care and Use Committee protocols.

### Tissue preparation

Fourteen μm thick coronal brain slices were prepared with a cryostat (Leica CM3050 S) and mounted onto microscope slides, and kept at -80°C until the day of receptor autoradiography.

### [^3^H] DAMGO binding

Binding was performed with the mu opioid receptor agonist D-Ala2,N,Me-Phe4, Gly-ol5-enkephalin (DAMGO) to quantify MOR expression in the brain. Slides were removed from -80°C and allowed to come to room temperature, and rapidly cool-aired dried before pre-incubation. Pre-incubation was performed at room temperature in a solution of 50mM Tris HCl with 0.9%NaCL for 30 minutes. Specific binding was performed in the same solution as the pre-incubation with the addition of 4nM [^3^H] DAMGO (PerkinElmer [Tyrosyl-3, 5-^3^H(N)]-DAMGO) for 60 minutes. Slides were then rinsed for 30 seconds in fresh ice-cold pre-incubation buffer solution, and finally rinsed once in distilled water at 4°C. Non-specific binding was determined in the presence of excess 10μM Naloxone (PerkinElmer [N-ALLYL-2,3-3H]-) in the same manner as specific binding.

Following receptor binding, slides were dried and exposed onto Kodak BioMax MR Film, alongside calibrated tritium standards (American Radiolabeled Chemical, St. Louis, MO.) for a period of 10 weeks. Films were then developed in a Kodak D-19 developer, dried, and scanned as a TIFF file at 1200dpi using a Brother MFC-J6510DW scanner.

### Regions of interest

**Fig 1:**
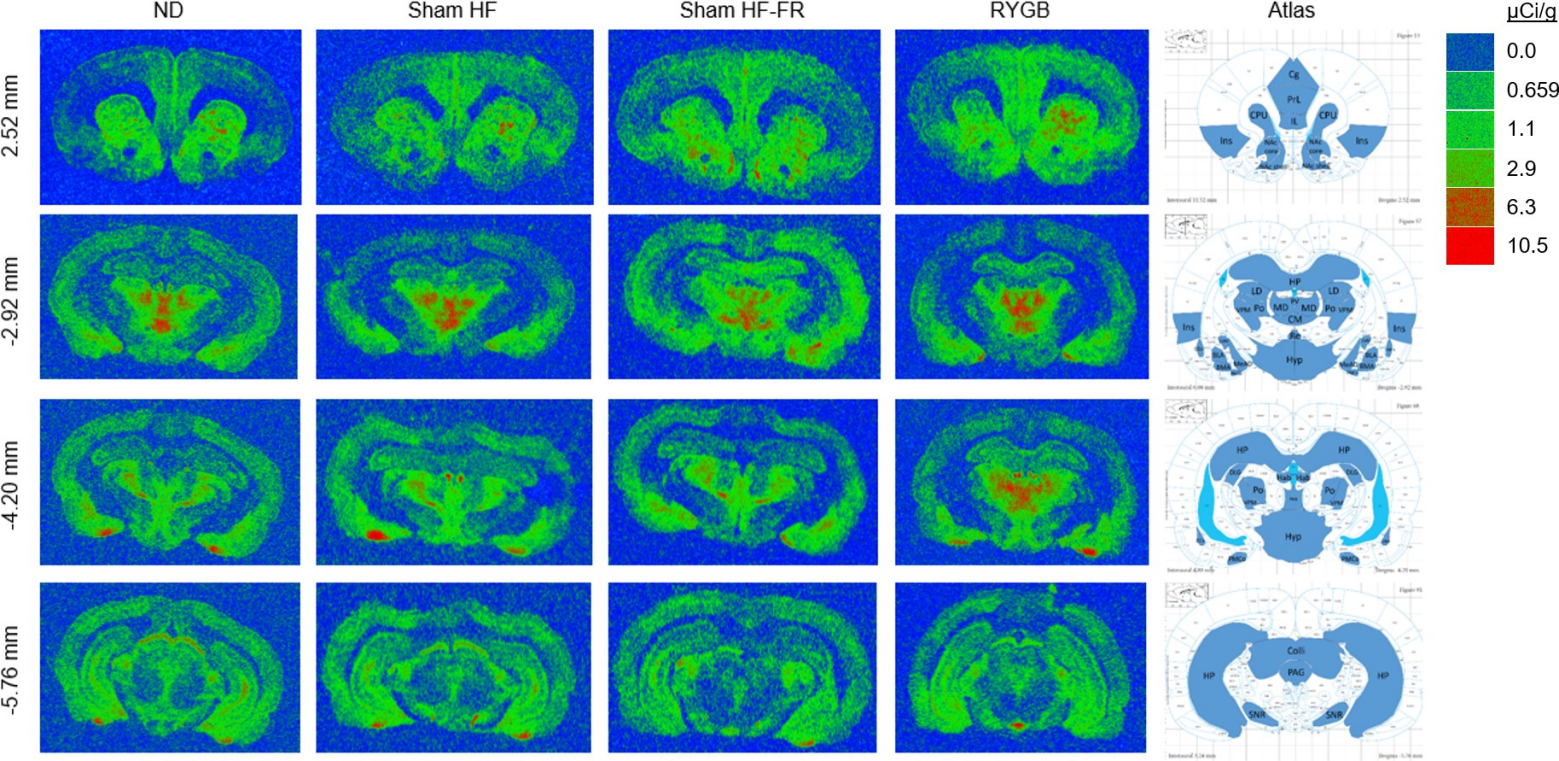
Representative images of [^3^H] DAMGO binding in brain regions of interest at four bregma levels. For ND (normal diet; no surgery), Sham (*ad libitum* high-fat diet; sham surgery), Sham FR (food restricted high-fat diet; sham surgery), and RYGB (*ad libitum* high-fat diet; RYGB surgery) rats. A quantitative binding scale of recorded standards is provided. Regions of interest are drawn on the atlas images adapted from the Paxinos and Watson rat brain atlas [36].

The scanned films were analyzed using ImageJ software (National Institutes of Health). The prelimbic cortex (PrL), infralimbic cortex (IL), cingulate cortex (Cg), insular cortex (Ins), habenula (Hab), hypothalamus (Hyp), basolateral amygdaloid nucleus (BLA), basomedial amygdaloid nucleus (BMA), central medial amygdaloid nucleus (CeM), posteromedial cortical amygdaloid nucleus (PMCo), periaqueductal gray (PAG), superior colliculus (Colli), dorsal endopiriform nucleus (DEn), dorsolateral striatum (DL CPU), dorsomedial striatum (DM CPU), ventrolateral striatum (VL CPU), ventromedial striatum (VM CPU), nucleus accumbens core (NAc core), nucleus accumbens shell (NAc Shell), and thalamic regions retrouniens area (Re), paraventricular thalamic nucleus (PV), central medial thalamic nucleus (CM), laterodorsal thalamic nucleus (LD), mediodorsal thalamic nucleus (MD), ventral posteromedial thalamic nucleus (VPM), posterior thalamic nuclear group (Po), and dorsal lateral geniculate nucleus (DLG) were examined for [^3^H] DAMGO binding (Fig 1).

### Statistics

All statistics were performed with Sigmaplot 11.0 (Systat Software, Inc., Chicago, Illinois). A one-way ANOVA was conducted for each region of interest to examine differences in binding between groups, with significance level set to *α* = 0.05. Post hoc tests were performed using Tukeys HSD where appropriate. Pearson correlations were run for all regions within each group to identify significant correlations between binding and body weight (*α* = 0.05).

## Results

### [^3^H] DAMGO binding

**Fig 2:**
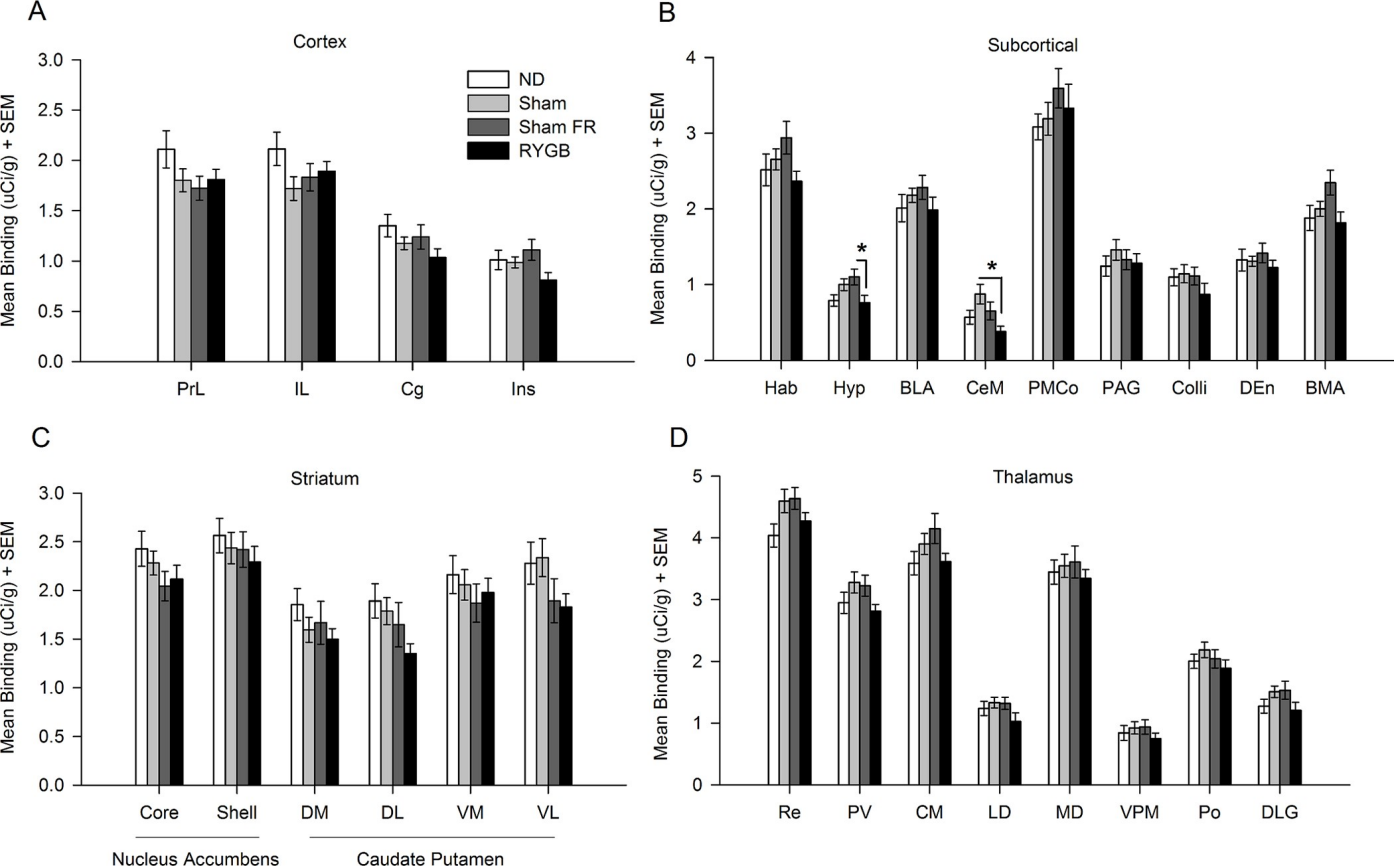
[^3^H] DAMGO binding within brain regions. Mean binding within the (**A**) prelimbic (PrL), infralimbic (IL), cingulate (Cg), and insular (Ins) cortices; (**B**) habenula (Hab), hypothalamus (Hyp), basolateral amygdaloid nucleus (BLA), central medial amygdaloid nucleus (CeM), posteromedial cortical amygdaloid nucleus (PMCo), periaqueductal gray (PAG), superior colliculus (Colli), dorsal endopiriform nucleus (DEn), and basomedial amygdaloid nucleus (BMA); (**C**) nucleus accumbens core (Nac core) and shell (Nac shell), dorsal medial (DMCPU), dorsal lateral (DLCPU), ventral medial (VMCPU), and ventral lateral (VLCPU) striatum; (**D**) retrouniens area (Re), paraventricular thalamic nucleus (PV), central medial thalamic nucleus (CM), laterodorsal thalamic nucleus (LD), mediodorsal thalamic nucleus (MD), ventral posteromedial thalamic nucleus (VPM), posterior thalamic nuclear group (Po), and dorsal lateral geniculate nucleus (DLG) is shown for ND (normal diet; no surgery), Sham (*ad libitum* high-fat diet; sham surgery), Sham FR (food restricted high-fat diet; sham surgery), and RYGB (*ad libitum* high-fat diet; RYGB surgery) rats. * *p* < .05.

A one-way ANOVA showed an effect of RYGB treatment within the Hyp (*F*(3,68) = 3.331; *p* < 0.05) and CeM (*F*(3,70) = 3.793; *p* < 0.05) (Fig 2). *Hyp*: RYGB group showed 30.66 ± 10.88% lower binding than Sham-FR group (*p* < 0.05). *CeM*: RYGB group showed 56.49 ± 10.36% lower binding than Sham-HF group (*p* < 0.01). No significant group differences were seen in cortical, striatal, or thalamic regions (Fig 2).

**Fig 3:**
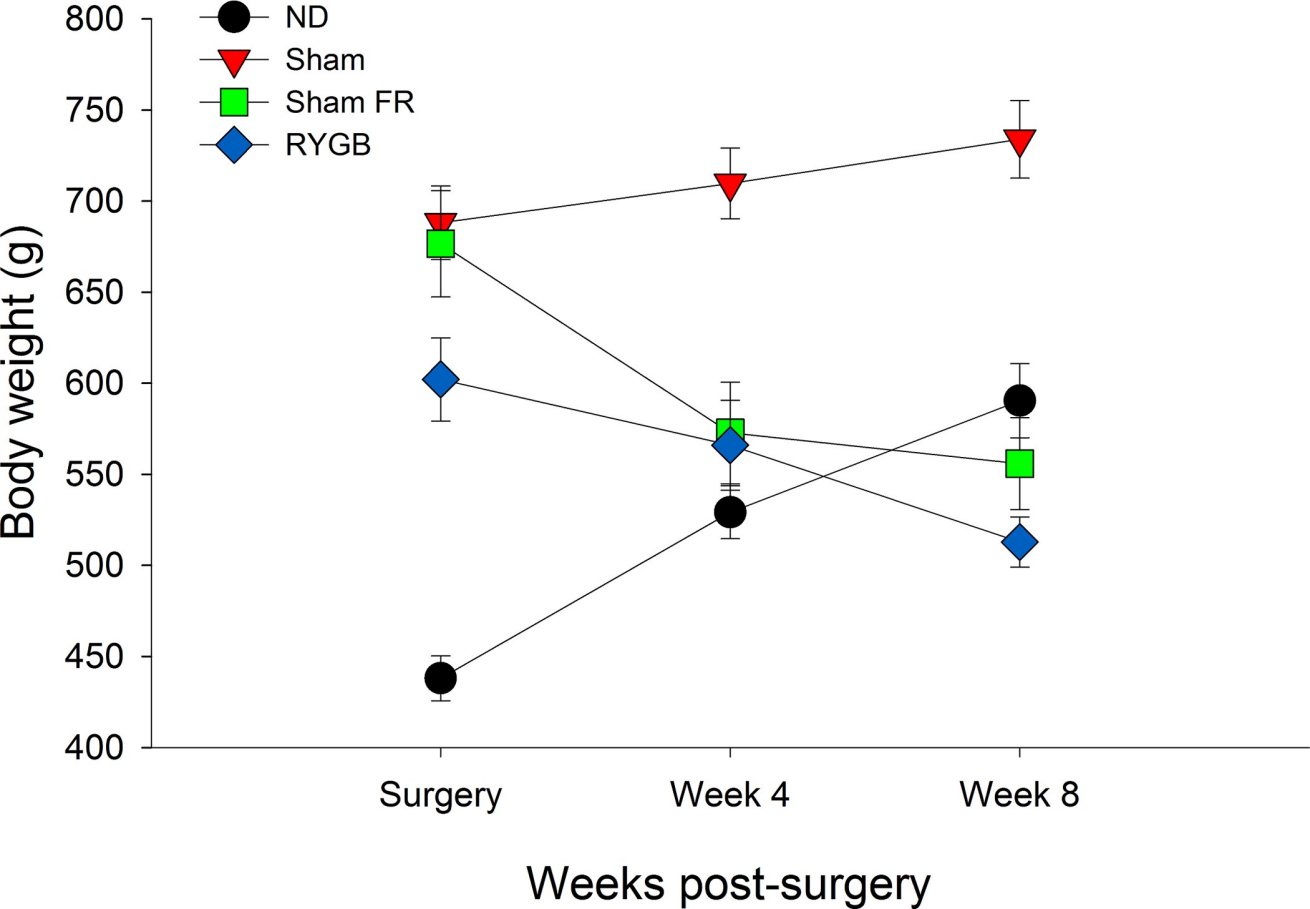
Body weight changes post-surgery. Weights were recorded day of surgery, 4 weeks after, and 8 weeks after (day of sacrifice) for ND (normal diet; no surgery), Sham (*ad libitum* high-fat diet; sham surgery), Sham FR (food restricted high-fat diet; sham surgery), and RYGB (*ad libitum* high-fat diet; RYGB surgery) rats.

Body weights were recorded at 12 weeks of age (day of surgery), 16 weeks (4 weeks post-surgery), and 20 weeks of age (8 weeks post-surgery) (Fig 3). A significant positive correlation between binding and body weight was identified in the PrL in the ND group (r = .962, *p* < .01). A significant negative correlation was found in the CeM in the Sham-HF group (r = -.652, *p* < .05). Significant positive correlations were found in the DMCPU (r = .683, *p* < .05), DLCPU (r = .698, *p* < .05), VMCPU (r = .736, *p* < .05), VLCPU (r = .721, *p* < .05), Hab (r = .769, *p* < .05), DEn (r = .702, *p* < .05), and Re (r = .668, *p* < .05) in the Sham-FR group. Significant negative correlations were found in the IL (r = -.892, *p* < .05) and Cg (r = -.970, *p* < .01) in the RYGB group.

The complete binding and bodyweight measurements underlying these results are available online (https://doi.org/10.17026/dans-22t-3tkd).

## Discussion

The results of this study show that RYGB surgery resulted in a significant 31% reduction in binding in the hypothalamus compared to animals on a food restricted HF diet, and a 56% reduction in binding in the central medial amygdala compared to the HF group. However, rats on a HF diet, either restricted or *ad libitum*, do not display changes in MOR binding compared to rats on a standard control diet.

The hypothalamus is involved in hunger and the energy regulation aspect of feeding [20]. Food deprivation has been shown to increase MOR mRNA expression in the ventral medial hypothalamus, arcuate nucleus, and lateral hypothalamus [37]. Food-deprived rats show preference for a high-fat diet over low-fat diet, but blocking of MORs with a selective antagonist reduces this preference. Moreover, stimulation of the paraventricular, dorsomedial, and lateral hypothalamus with MOR agonists in sated rats has been shown to induce strong feeding responses [38]. This indicates that increased opioid signaling in the hypothalamus can override the energy-regulation aspects of food consumption to encourage binge eating even when the subject is sated. It has also been reported that MOR inhibition in the lateral hypothalamus, but not the nucleus accumbens or ventral tegmental area, reduces food intake in food-deprived mice, suggesting that opioid signaling in the hypothalamus mediates hunger rather than the rewarding aspect of food consumption [39]. Our laboratories previously reported that body weight significantly decreases in the 8 weeks following RYGB surgery [34] (Fig 3). The reduction in hypothalamus [^3^H] DAMGO binding seen between the high-fat fed Sham-FR and RYGB groups suggests that decreased body weight may be due to decreased hunger driven by downregulated mu opioid signaling in this region. Activity of the proopiomelanocortin (POMC) neurons of the arcuate nucleus suppresses food intake through melanocortin signaling [40], and can be inhibited by local MOR binding [41]. Additionally, melanocortin-4 receptor signaling is necessary for weight loss following RYGB [42, 43]. It’s possible that RYGB surgery modifies MOR expression in the POMC neurons of the hypothalamus to reduce consumption to accommodate for the decreased stomach volume, although the effect of decreased stomach volume on long-term weight loss is debated [44].

The central amygdala triggers a motivated response to learned cues, either aversive or appetitive, and has been suggested to influence food preferences [45–47]. The level of motivation is dependent on the state of the mesolimbic dopamine circuits and central amygdala opioid signaling at the time of the encounter [47]. Opioid signals specifically are important for generating a ‘wanting’ in response to learned cues. MOR agonists have previously been shown to increase food intake when injected into the amygdala [20, 48, 49]. This injection increased directed ‘wanting’ but decreased hedonic ‘liking’ in a taste reactivity test. As such, increased MOR binding encourages pleasure-seeking behavior. The reduced [^3^H] DAMGO binding we observed in the central medial amygdala in the RYGB group compared to unrestricted HF diet group then suggests decreased ‘wanting’ for food, which we expect would mediate lower consumption. This is supported by a study showing decreased preferred diet intake following injection of the MOR antagonist naltrexone into the central amygdala [50]. This does not account for the increase in addictive behavior seen in some RYGB patients. However, opioid signaling in the central amygdala also plays a role in the stress response. Micro-injections of DAMGO into the rat central amygdala have been shown to induce distinct behavioral changes in response to potential and discrete threats [51]. Particularly relevant for SUD, the central amygdala emerged as an integrative hub for anxiety and alcohol use disorders [52]. Therefore, increased sensitivity to stress as a consequence of reduced MOR in the CeM may contribute to the alcohol misuse, as well as increased incidence of accidents and suicide following RYGB surgery [53, 54].

Neuropeptide Y (NPY) and corticotropin releasing hormone (CRH) are both synthesized in the paraventricular nucleus of the hypothalamus and are associated with the stress response [55, 56]. Dysregulation of these neuropeptides is associated with depression, and reduced plasma levels of both NPY and CRH have been found in suicidal individuals [57]. What’s more, the MOR can indirectly inhibit hypothalamic CRH-releasing neurons [58]. If increased stress sensitivity is responsible for the increased incidence of suicide following RYGB surgery, one would expect expression of these peptides to be reduced in RYGB patients. One study in rats showed a significant reduction in NPY expression in the hypothalamus 10 days following RYGB [59]. On the other hand, another group reported elevated CRH levels in RYGB rats [60]. Additionally, a reduction in plasma NPY levels following RYGB has not been seen clinically [61, 62]. Further work is needed to determine the reason for this discrepancy in stress factor levels between species following RYGB.

The limited change in [^3^H] DAMGO binding between experimental groups is somewhat surprising given results of previous studies. Using PET with [^11^C]carfentanil in humans, one group showed that obese subjects had reduced MOR availability in nearly all brain regions associated with feeding, including the ventral striatum, insula, amygdala, and thalamus [63]. MOR availability rose significantly in all these areas following gastric bypass surgery, to levels comparable to those in lean patients. Furthermore, BMI was negatively correlated with MOR availability in every brain region except the cingulate cortex. None of these changes were identified as significant in our study between any treatment groups, apart from the central medial amygdala. However, the changes in receptor availability seen in these PET studies does not necessarily imply changes in receptor levels. Obesity may promote a change in basal neurotransmitter levels without affecting changes in receptor expression itself. Obesity has also been shown to have differential effects on dopamine D2-type receptor mRNA and protein expression compared to binding [64]. Though it is unclear why changes were only seen in the RYGB group within the hypothalamus and central amygdala. It must also be noted that there may be species differences in feeding-induced opioid signaling. Whereas consumption of a palatable meal induces opioid release in the hypothalamus, anterior cingulate cortex, and nucleus accumbens in rats [65, 66], opioid release in the ventral striatum, thalamus, and anterior cingulate cortex has been reported in humans following consumption of palatable food [67].

Another study using DAMGO autoradiography reported increased binding in the basolateral and basomedial amygdala, habenula, and dentate gyrus in rats fed a highly palatable diet [28]. These results were not confirmed by our study. Surprisingly, we also identified no significant changes in [^3^H] DAMGO binding in the nucleus accumbens between groups, a region highly susceptible to increased consumption of a palatable diet in response to stimulation with DAMGO [27]. Previous studies have reported decreased mRNA expression for the mu opioid receptor in this region following consumption of a highly palatable diet [23], but this did not seem to translate to a decrease in mu-opioid receptor protein levels in the present study.

We previously reported that dopamine receptor expression decreases in the nucleus accumbens and striatum of high-fat diet-fed rats, and returns to control levels following RYGB surgery [34]. Increased dopamine signaling in this region can facilitate reward-seeking behavior including food and drug intake [68]. Injection of DAMGO into the paraventricular nucleus of the hypothalamus has been shown to increase dopamine release in the NAc, suggesting that mu opioid signaling in the hypothalamus contributes to NAc dopamine release in a way that might stimulate appetitive behavior [69]. However, we have shown that hypothalamus MOR levels decreased while NAc dopamine receptor levels increased in HF diet-fed rats following RYGB surgery, failing to support this conclusion. Alternatively, a hypoactive dopaminergic system can result in decreased pleasure in response to normal stimuli, and thus abnormal craving behavior for drugs of abuse and binge eating [70]. This decreased dopamine transmission is characteristic of a hyposensitivity to reward which has been described as Reward Deficiency Syndrome (RDS) [70]. If RYGB surgery modulates NAc dopamine signaling through decreased mu opioid transmission in the hypothalamus, a reward-deficient subject may be more sensitive to the diminished signaling post-surgery. Alcohol, cocaine, opioids, and nicotine use all stimulate dopamine transmission in the brain [70, 71]. Similarly, reduced MOR signaling may cause increased opioid intake to compensate. Rats receiving RYGB display higher morphine self-administration rates than lean, obese, and caloric intake-matched counterparts [16]. The downregulated MOR signaling reported here and by others supports this notion [33]. No studies have of yet investigated the effect of administered MOR agonists or antagonists on feeding or motivated behavior following RYGB, which could provide further insight into the induced changes in reward signaling.

Taken together, our results show no changes in MOR levels following *ad libitum* or restricted high-fat diet, with significant decreases in mu-opioid signaling in specific regions mediating energy regulation (hypothalamus) and stress (central medial amygdala) following RYGB surgery. Further work is needed to determine whether the changes in MOR levels in the RYGB group translate to increased addictive behavior by a subpopulation of subjects seen clinically.
